# Knowledge and Preventive Practices Regarding Cervical Cancer Among Women in Nkoranza South Municipality, Ghana: A Cross-Sectional Study

**DOI:** 10.7759/cureus.108654

**Published:** 2026-05-11

**Authors:** Emmanuel S Aboagye, Uchechukwu B Abioke, Chinedu E Ogugua, Felix Danquah, Oyinlade C Ogundare, Lilian O Okwuonu

**Affiliations:** 1 Physician Assistantship, Anglican University College of Technology, Nkoranza, GHA; 2 Physical Therapy, University of Benin Nigeria, Benin, NGA; 3 Obstetrics and Gynecology, Aminu Kano Teaching Hospital, Kano, NGA; 4 Public Health, Kwame Nkrumah University of Science And Technology, Kumasi, GHA; 5 Chemical Sciences, Lagos State University of Science and Technology, Lagos, NGA; 6 Nursing, Wayne County Community College District, Detroit, USA

**Keywords:** attitudes, cervical neoplasms, cross-sectional studies, health knowledge, human papillomavirus vaccines, mass screening, papanicolaou test, practice, risk factors

## Abstract

Introduction: Cervical cancer remains a major public health challenge in low- and middle-income countries, including Ghana. Despite the availability of effective preventive measures, the incidence and mortality rates remain high. The aim of the study was to assess cervical cancer awareness, knowledge of risk factors, screening practices, and human papillomavirus (HPV) vaccination awareness and uptake among sampled women in Nkoranza South Municipality, Bono East Region, Ghana.

Methods: A descriptive cross-sectional study was conducted between January and June 2023 among women aged between 18 and 45 years in the Nkoranza South Municipality, Bono East Region, Ghana. Data were collected using a structured questionnaire administered in English or orally in the Bono language when necessary. Convenience sampling was used to recruit the participants among the eligible populations in the municipality. The questionnaire was dichotomous and multiple-choice and included socio-demographic variables, cervical cancer awareness, knowledge of risk factors, HPV vaccination awareness and uptake, screening practices, and barriers to prevention. Descriptive statistics were used to summarize data, which were presented in tables, graphs, and charts.

Results: The analysis included 70 women who filled out the questionnaire. Overall, 65 (92.9%) respondents were aware of cervical cancer. Among the 65 respondents who had heard of cervical cancer, 49 (75.4%) believed that cervical cancer could be prevented, 27 (41.5%) had heard of the HPV vaccine, and 15 (23.1%) had ever been screened. Overall, these findings show that although awareness of cervical cancer was high, knowledge of specific preventive measures and uptake of screening remained low.

Conclusion: Although cervical cancer awareness was high among the study participants, this was not accompanied by similarly high uptake of screening or HPV vaccination. These findings suggest an awareness-practice gap among the sampled women and highlight the need for structured health education that promotes practical knowledge of screening and vaccination services.

## Introduction

Despite being largely preventable through screening and vaccination, cervical cancer remains the fourth most common cancer among women worldwide [1]. In the year 2020 alone, 604,127 women were estimated to have the disease [2]. Of this, 341,831 were estimated to have died the same year. This is about 13 new cases and seven deaths per 100,000 women annually at the global level, but these aggregate numbers mask significant regional variations [1]. Incidence is also as low as two per 100,000 women in certain countries, but it increases to over 80 per 100,000 in others [2]. The same applies to mortality, with a range of about one per 100,000 women in a high-income country (HIC) like Switzerland to more than 50 per 100,000 in such places as Eswatini [3].

This pattern is not coincidental. It closely follows lines of development and access. The incidence in countries with low human development is some three times higher, and mortality is up to six times higher, than in highly developed countries [2]. Such inequalities indicate the unequal access to the preventive measures, as well as the variation in the level of public awareness and health-seeking behaviour [4]. In HICs, the incidence and mortality related to this disease have decreased significantly through organised screening and extensive vaccination, but low- and middle-income nations are still forced to bear a disproportionate burden of disease.

Cervical cancer is one of the most common causes of cancer-related morbidity and mortality in Ghana, with an estimated 3,000 new cases and close to 1,700 deaths every year [5]. The age-standardised incidence rate stands at about 27 per 100,000 women, indicating a high burden in the country that has remained high [5]. Despite preventive measures that have been introduced in the National Reproductive Health Policy, including visual inspection with acetic acid (VIA) screening and human papillomavirus (HPV) vaccination, there is still low coverage of these services. Access to screening facilities, poor health education, culture, and poor awareness remain the barriers to successful prevention [6].

Cervical cancer develops over a prolonged period, typically 15 to 20 years, with persistent infection by high-risk HPV types being the primary underlying cause. Although HPV infection is mostly temporary, persistent infection could result in precancerous lesions, which can further develop into invasive cancer with no treatment [7]. This long natural history offers a significant chance of early diagnosis and intervention in the form of screening. In other regions of Ghana, research indicates an inconsistent knowledge level and uptake of screening, which underscores the existing knowledge-practice gaps [8]. 

Nonetheless, there is limited information on cervical cancer awareness, knowledge of risk factors, screening practices, and HPV vaccination awareness and uptake among sampled women in Nkoranza South Municipality. The aim of this study, therefore, was to assess cervical cancer awareness, knowledge of risk factors, screening practices, and HPV vaccination awareness and uptake among sampled women in Nkoranza South Municipality, Bono East Region, Ghana.

## Materials and methods

Study design and setting

A descriptive cross-sectional study was conducted from January to June 2023 among women in the Nkoranza South Municipality, Bono East Region, Ghana. The study aimed to assess knowledge and preventive practices regarding cervical cancer.

Study population and eligibility criteria. Participants were recruited through a door-to-door approach within communities in Nkoranza South Municipality. Women aged between 18 and 45 years were eligible to participate. Women were excluded if they declined to provide informed consent or had a prior diagnosis of cervical cancer. No additional exclusion criteria were applied.

Sample size

A total of 70 women were recruited for this study. No formal sample size calculation was conducted because the study was exploratory, and there was no locally specific prevalence estimate for cervical cancer knowledge or screening uptake in Nkoranza South Municipality at the time of study planning. The sample size was therefore determined pragmatically based on the study period, available field resources, accessibility of eligible participants, and the feasibility of completing door-to-door recruitment within the municipality between January and June 2023. Efforts were made to approach all eligible and consenting women encountered during the recruitment period.

Sample technique and procedure

A non-probability sampling technique, specifically convenience sampling, was used because there was no complete sampling frame and the study was exploratory and resource-limited [9]. Participants were recruited through a door-to-door approach in selected communities within Nkoranza South Municipality. The research team approached households, explained the purpose of the study, and screened women for eligibility. Women were eligible if they were aged between 18 and 45 years, lived in the municipality, and provided informed consent. Women who declined consent or reported a previous diagnosis of cervical cancer were excluded. Eligible women who agreed to participate completed the questionnaire immediately after consent was obtained.

Data collection

Data were collected using a structured, closed-ended questionnaire developed after reviewing previous literature on cervical cancer knowledge, risk factors, HPV vaccination, and screening practices. The questionnaire was organised into sections covering socio-demographic characteristics, awareness of cervical cancer, knowledge of risk factors, awareness of screening methods, screening uptake, HPV vaccination status, and barriers to prevention.

The questionnaire was prepared in English and administered in English or orally in the Bono language, where necessary to support participant understanding. Before final data collection, the questionnaire was pre-tested among a small group of women with characteristics similar to the target population. The pre-test participants were not included in the final study sample. Feedback from the pre-test was used to revise unclear wording, improve relevance, and ensure that the questions were culturally appropriate and understandable in both English and Bono.

No formal psychometric validity or reliability testing was conducted because the questionnaire mainly contained descriptive, closed-ended knowledge and practice items rather than scale-based constructs. Research assistants involved in data collection were trained on the study objectives, eligibility criteria, informed consent process, questionnaire administration, and confidentiality procedures. Completed questionnaires were reviewed daily for completeness and consistency. Incomplete or unclear responses were checked during data collection, where possible. Data were entered into Microsoft Excel (Microsoft Corp., Redmond, WA, USA) and cross-checked against the completed questionnaires to minimise entry errors before analysis in JASP version 0.17.2.1 (JASP Team, University of Amsterdam, Amsterdam, Netherlands). Frequencies and percentages were recalculated during analysis to ensure consistency between the tables and narrative results. Appendix A shows the questionnaire that was used to collect the data.

Data analysis

Data were analysed using Microsoft Excel 2019 and JASP version 0.17.2.1 for descriptive statistical analysis. Descriptive statistics, including frequencies, percentages, means, and standard deviations, were used to summarise variables. Results were presented in tables and charts for clarity.

Ethical considerations

Ethical oversight for this study was provided through the institutional research review process at the Anglican University College of Technology, Nkoranza Campus, Ghana. The institution did not operate a formal Institutional Review Board at the time of the study. In its absence, the research protocol was reviewed and approved by the University College Research Review Panel on February 23, 2023, before data collection commenced. No formal approval number was issued. Permission to conduct the study was also obtained from the Nkoranza South Municipal Directorate.

This was a descriptive, non-interventional study involving minimal risk to participants. Participation was voluntary, and all participants received information about the study purpose, confidentiality, and their right to withdraw at any time without consequence. Written informed consent was obtained from all participants before inclusion. No personally identifying information was included in the analysis. Data were handled confidentially and used only for the purpose of the study. The study was conducted in accordance with internationally recognised ethical principles for research involving human participants, consistent with the Declaration of Helsinki [10].

## Results

A total of 70 women participated in the study. The largest proportion of participants was aged between 18 and 25 years (28, 40.0%). Most respondents had attained a tertiary education (53, 75.7%). In terms of occupation, the largest proportion were students (30, 42.9%), while a small proportion were unemployed (three, 4.3%). Regarding marital status, the majority were single (44, 62.9%). Table 1 presents the socio-demographic characteristics of the study participants.

**Table 1:** Socio-demographic characteristics of the study participants (n = 70)

Variables	Category	Frequency (n = 70)	Percentage (%)
Age (years)	18–25	28	40
	26–32	18	25.7
	33–40	13	18.6
	41–45	11	15.7
Educational status	No formal schooling	2	2.9
	Primary (Classes 1–6)	1	1.4
	Junior high school	3	4.3
	Senior high school	11	15.7
	University/College	53	75.7
Occupation	Unemployed	3	4.3
	Student	30	42.9
	Self-employed	18	25.7
	Employed	19	27.1
Marital status	Single	44	62.9
	Married	19	27.1
	Divorced	2	2.9
	Separated	5	7.1

As shown in Table 2, 65 (92.9%) of the 70 respondents had heard of cervical cancer, while five (7.1%) had not. Among the 65 respondents who had heard of cervical cancer, television and radio were the most commonly reported sources of information (39, 60.0%), followed by school (13, 20.0%), and other platforms (13, 20.0%).

**Table 2:** Respondents' awareness of cervical cancer and source of information. Awareness of cervical cancer was calculated out of the total sample, n = 70. The source of information was calculated only among respondents who had heard of cervical cancer, n = 65. Percentages for the source of information sum to 100%.

Variable	Category	Frequency, n	Percentage, %
Awareness of cervical cancer	Yes	65	92.9
Awareness of cervical cancer	No	5	7.1
Source of information	School	13	20
Source of information	TV/Radio	39	60
Source of information	Other platforms	13	20

Table 3 presents respondents’ knowledge of the causes, risk factors, and prevention of cervical cancer among the 65 respondents who confirmed awareness. The findings indicate varying levels of understanding, with respondents demonstrating awareness of key risk factors, such as infection and multiple sexual partners, while demonstrating uncertainty regarding other risk factors and preventive measures.

**Table 3:** Distribution of respondents based on their knowledge of the causes, associated risk factors, and preventive measures of cervical cancer. All percentages are calculated out of n=65 (respondents who confirmed awareness of cervical cancer). Row percentages sum to 100%. STI: sexually transmitted infection

Variable	Yes n (%)	No n (%)	Maybe n (%)
Infection causes cervical cancer	56 (86.2)	2 (3.1)	7 (10.8)
Multiple sexual partners increase risk	52 (80.0)	4 (6.2)	9 (13.8)
Early sexual intercourse increases risk	39 (60.0)	8 (12.3)	18 (27.7)
STI history increases risk	52 (80.0)	13 (20.0)	0 (0.0)
Early sex (≤16 years) is risky	53 (81.5)	7 (10.8)	5 (7.7)
Smoking increases risk	40 (61.5)	24 (36.9)	1 (1.5)
Alcohol increases risk	25 (38.5)	24 (36.9)	16 (24.6)
Cervical cancer is preventable	49 (75.4)	2 (3.1)	14 (21.5)

As shown in Table 3, the majority of respondents identified infection as a cause of cervical cancer (56, 86.2%) and most recognised multiple sexual partners (52, 80.0%) and a history of sexually transmitted infections (52, 80.0%) as risk factors. A similarly high proportion considered early sexual intercourse at or below 16 years to be risky (53, 81.5%), though fewer respondents associated early sexual intercourse in general with increased risk (39, 60.0%). Awareness of lifestyle-related risk factors was comparatively lower, with 40 (61.5%) identifying smoking and only 25 (38.5%) identifying alcohol consumption as contributing factors. Regarding prevention, 49 (75.4%) of respondents believed that cervical cancer could be prevented, while 14 (21.5%) were uncertain.

**Figure 1:**
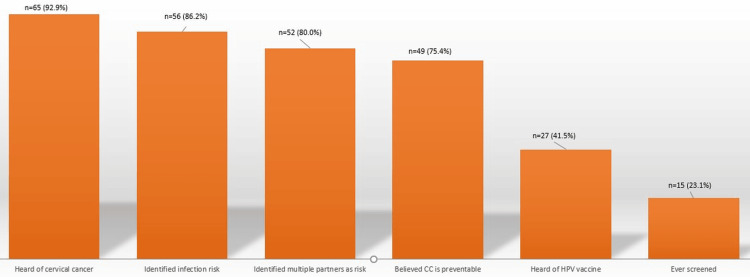
Key indicators of cervical cancer awareness, knowledge, and preventive practices among respondents (n=65). Values above the bars represent percentages. “Heard of cervical cancer” is calculated out of the total sample (n=70); all other indicators are calculated out of n=65. CC: cervical cancer; HPV: human papillomavirus

Figure 1 illustrates key indicators of respondents’ awareness, knowledge, and preventive practices regarding cervical cancer. The figure is based on the 65 respondents who confirmed awareness of cervical cancer, excluding the five who reported no prior awareness. The chart highlights high levels of general awareness, moderate knowledge of risk factors and prevention, and comparatively low levels of vaccination awareness and screening practices.

Table 4 presents respondents' knowledge and practices regarding cervical cancer vaccination. Although 65 (92.9%) of respondents were aware of cervical cancer, only 15 (23.1%) had ever been screened. Awareness of the HPV vaccine was reported by 27 (41.5%) of respondents, and only four (6.2%) had ever been vaccinated. Knowledge of screening modalities was similarly limited, with 35 (53.8%) aware of Pap smears, nine (13.8%) aware of the HPV test, and 21 (32.3%) having no knowledge of any screening method. The findings reveal generally low awareness of the HPV vaccine, limited knowledge of its availability, and very low uptake among respondents. All items in Table 4 were administered to the 65 respondents who confirmed awareness of cervical cancer.

**Table 4:** Respondents’ knowledge and practices regarding cervical cancer vaccination. All percentages are calculated out of n=65 (respondents who confirmed awareness of cervical cancer). HPV: human papillomavirus

Variable	Yes n (%)	No n (%)	Maybe n (%)
Heard of the HPV vaccine	27 (41.5)	30 (46.2)	8 (12.3)
Vaccine available in Ghana	25 (38.5)	13 (20.0)	27 (41.5)
Know where to get vaccinated	26 (40.0)	39 (60.0)	0 (0.0)
Ever vaccinated	4 (6.2)	61 (93.8)	0 (0.0)
Can die from cervical cancer	58 (89.2)	1 (1.5)	6 (9.2)
Ever screened	15 (23.1)	50 (76.9)	0 (0.0)
HPV test known	9 (13.8)	56 (86.2)	0 (0.0)
Pap smear known	35 (53.8)	30 (46.2)	0 (0.0)
No idea about screening	21 (32.3)	44 (67.7)	0 (0.0)

## Discussion

The findings of this study indicate that although the sampled women had high awareness of cervical cancer, this was not consistently reflected in screening and vaccination practices. Despite high awareness (65, 92.9%), uptake of screening remained disproportionately low (15, 23.1%). A significant number of the respondents could name major risk factors, including infection (56, 86.2%) and more than one sexual partner (52, 80.0%). Nonetheless, this knowledge was not consistent in other areas, with a significant lack of certainty about aspects such as early sexual activity, smoking, and alcohol consumption. This suggests that while basic awareness of cervical cancer exists, significant knowledge gaps remain. This is in line with a study conducted in South Africa, which showed that while female students were very aware of cervical cancer, the knowledge of the respective risk factors and prevention strategies was incomplete [11].

The results also indicate that the informal and media sources were the major sources of awareness, especially the television and radio. Although these platforms effectively create general awareness, the knowledge differences among particular risk factors imply that the information shared might lack the depth and clarity necessary to guide to more profound knowledge. This trend aligns with findings from low- and middle-income settings, where knowledge is often widespread but insufficient to drive behavioural change [12].

There is a strong disparity between knowledge of the seriousness of the disease and preventive measures. Even though the majority of respondents (58, 89.2%) were aware that cervical cancer is a potentially deadly disease, only 15 (23.1%) had ever been screened. There was also limited awareness of screening modalities, with only 35 (53.8%) of the respondents having knowledge of Pap smears and a lower percentage having awareness of HPV tests, with 21 (32.3%) having no knowledge of any screening modality. This suggests a gap between general awareness of cervical cancer and practical knowledge or uptake of available preventive services among the study participants.

In Nigeria, the same trends have been noted, with the screening uptake being low even though there is a certain degree of awareness [13]. The same trend can be observed regarding vaccination. Although 49 (75.4%) of respondents who had heard of cervical cancer believed that cervical cancer is preventable, awareness of the HPV vaccine (27, 41.5%) and vaccine uptake (four, 6.2%) remained low. Moreover, over two-thirds of the respondents, 39 (60.0%), were unaware of where they could obtain vaccination services. This suggests that believing cervical cancer is preventable was not consistently accompanied by awareness or uptake of specific preventive measures, such as HPV vaccination and screening, among the study participants. The same has been observed in Ghana, where women showed a lack of awareness regarding HPV vaccination and low uptake, though there was awareness regarding cervical cancer [8].

There are a number of limitations in this study. The findings describe observed patterns but cannot establish causation. The data were self-reported and could be prone to recall bias or social desirability bias, especially when it comes to sensitive issues like screening history and vaccination status. The research was done in one municipality with the help of a convenience sample. The sample was young, predominantly single, and highly educated, with 53 (75.7%) of the respondents having completed tertiary education. This does not reflect the general female population in Ghana and probably overestimates the level of knowledge as compared to the general population. Consequently, the results may not be applicable to other populations that have different demographic or socio-cultural data. Moreover, although the research found a lack of screening and vaccination, it did not delve deeper into the factors that shape these trends, such as cultural beliefs, financial constraints, or constraints of the health system itself. A qualitative study would be of great help in understanding these underlying factors further. Another limitation is that the institution did not have a formal institutional review board at the time of the study. Although the research protocol was reviewed and approved through the University College Research Review Panel before data collection, the absence of a formal institutional review board process may limit the strength of the ethical oversight documentation available for the study. Despite these limitations, the study provides useful preliminary local evidence on the gap between awareness and preventive action among the study participants.

In general, the results indicate that the awareness of cervical cancer among the respondents does exist, but it is not evenly distributed. Most awareness is with the widely spread messages, whereas there are gaps in the areas that need more detailed or practical knowledge. These findings suggest that future interventions may need to move beyond general awareness and provide practical information on where and how women can access screening and HPV vaccination services. In similar low-resource settings, mobile health interventions, including reminders and health education messages, have been explored as possible strategies to support cervical cancer screening uptake [14]. However, further research would be needed to evaluate the effectiveness of such approaches in Nkoranza South Municipality.

## Conclusions

Among the sampled women, the findings suggest that cervical cancer prevention efforts should extend beyond general awareness and include structured health education on screening and vaccination. While mass media (television and radio) can raise awareness, they may lack the detail required to promote screening and vaccination. These gaps may be addressed through context-appropriate strategies such as mobile health reminders, health education messages, social media-based outreach, and low-cost printed materials, although further studies are needed to evaluate their effectiveness in this setting. Low-cost, low-technology approaches, such as posters and other printed materials in tro-tros (public minibuses), markets, and health centres, can act as a constant reminder and reach women who may not seek formal health services.

At a systems level, routine reproductive health visits could provide opportunities to promote screening and vaccination. Greater coordination between the Ghana Health Service and the Ministry of Health may also improve the visibility of prevention services. A cost-effectiveness analysis of cervical cancer screening and vaccination in Ghana would also guide resource prioritisation. Finally, future research should explore how cultural and traditional beliefs influence cervical cancer prevention and care to inform behavioural interventions that extend beyond knowledge.

## Appendices

Appendix A

Structured Questionnaire on Knowledge, Risk Factors, and Preventive Practices Regarding Cervical Cancer

**Table 5:** Questionnaire on demographic characteristics and knowledge STI: sexually-transmitted infections

Section	Variable	Question Item	Response Options
I. Demographic Characteristics	Age	What is your age?	18-25, 26-32, 33-40, 41-45
	Marital Status	What is your marital status?	Single, Married, Separated, Divorced
	Education Level	What is your highest level of education?	No formal education, Primary, Secondary, University/College
	Occupation	What is your current occupation?	Self-employed, Employed, Unemployed, Student
II. Knowledge and Risk Factors	Awareness	Have you ever heard of cervical cancer?	Yes, No, Maybe
	Source	Where did you first hear about cervical cancer?	School, TV/Radio, Other
	Mortality	Can cervical cancer lead to death?	Yes, No, Maybe
	Infection	Is cervical cancer associated with an infection?	Yes, No, Maybe
	Multiple Partners	Does having multiple sexual partners increase risk?	Yes, No, Maybe
	Early Sex	Does early sexual intercourse increase risk?	Yes, No, Maybe
	Prevention	Is there an effective method that reduces risk?	Yes, No, Maybe
	Susceptibility	Do you think you could develop cervical cancer?	Yes, No, Maybe
	Smoking	Does smoking contribute to cervical cancer?	Yes, No, Maybe
	Alcohol	Does alcohol contribute to cervical cancer?	Yes, No, Maybe
	STI	Does STI history increase risk?	Yes, No, Maybe

**Table 6:** Questionnaire on vaccination and screening HPV: human papillomavirus

Section	Variable	Question Item	Response Options
III. Vaccination and Screening	Vaccine Awareness	Have you heard about the HPV vaccine?	Yes, No, Maybe
	Availability	Is the HPV vaccine available in Ghana?	Yes, No, I don't know
	Effectiveness	Does it provide 100% protection?	Yes, No, I don't know
	Access	Do you know where to get vaccinated?	Yes, No
	Vaccination Status	Have you been vaccinated?	Yes, No, I don't know
	Screening	Have you been screened?	Yes, No
	Methods	Which screening methods do you know?	Pap smear, HPV test, I don't know
